# 

**Published:** 1858-10

**Authors:** 


### THE SANITARY REVIEW.

The Death-Rate of England . . . . . . 209

Idiotcy—its Why and Wherefore . . . . . 225

Some Account of a Swine Pestilence . . . . . 234

Sanitary Science: Past and Present . . . . . 245

**EPITOME OF SANITARY LITERATURE:**

Lindsay. Transmission of Disease . . . . 246

Davis. Exercise for Consumptives . . . . 247

Guy. Compositors and Consumption . . . . 248

Nash. Cholera and Juggernaut . . . . . 248

Murchison. Type of Continued Fever . . . . 249

Chemical Manufactories and Public Hygiene . . . 250

Miscellaneous Works . . . . . . 251

**ORIGINAL COMMUNICATIONS:**

Epidemics and their every day Causes. By W. I. Cox, Esq. . . 254

An Ague Endemic at Cannington. By Alfred Haviland, Esq. . . 266

**SANITARY AND SOCIAL SCIENCE.**

Constantinople. By R. F. Foote, M.D. . . . . 270

Sewage and Sewer Gases. By H. Letheby, M.B. . . . 275

**PROGRESS OF EPIDEMICS in Forty-two Towns and Districts, contributed specially, by the Staff of Local Observers** . . . . 296

**SANITARY LEGISLATION** . . . . . . 314

**TRANSACTIONS of the EPIDEMIOLOGICAL SOCIETY of LONDON:**

On the Localized causes of Cholera. By F. H. Johnson, Esq. . . 53

On the Difficulties of the Study of prevailing Diseases. By W. H. Michael, Esq., Mayor of Swansea . . . . 65

Quarterly Report of Proceedings . . . . . 72

